# Fine Mapping of the Barley Chromosome 6H Net Form Net Blotch Susceptibility Locus

**DOI:** 10.1534/g3.116.028902

**Published:** 2016-04-19

**Authors:** Jonathan Richards, Shiaoman Chao, Timothy Friesen, Robert Brueggeman

**Affiliations:** * Department of Plant Pathology, North Dakota State University, Fargo, North Dakota 58108-6050; † United States Department of Agriculture-Agricultural Research Service (USDA-ARS), Red River Valley Agricultural Research Center, Cereal Crops Research Unit, Fargo, North Dakota, 58102-2765

**Keywords:** high-resolution mapping, inverse gene-for-gene, necrotrophic specialist

## Abstract

Net form net blotch, caused by the necrotrophic fungal pathogen *Pyrenophora teres* f. *teres*, is a destructive foliar disease of barley with the potential to cause significant yield loss in major production regions throughout the world. The complexity of the host–parasite genetic interactions in this pathosystem hinders the deployment of effective resistance in barley cultivars, warranting a deeper understanding of the interactions. Here, we report on the high-resolution mapping of the dominant susceptibility locus near the centromere of chromosome 6H in the barley cultivars Rika and Kombar, which are putatively targeted by necrotrophic effectors from *P. teres* f. *teres* isolates 6A and 15A, respectively. Utilization of progeny isolates derived from a cross of *P. teres* f. *teres* isolates 6A × 15A harboring single major virulence loci (*VK1*, *VK2*, and *VR2*) allowed for the Mendelization of single inverse gene-for-gene interactions in a high-resolution population consisting of 2976 Rika × Kombar recombinant gametes. *Brachypodium distachyon* synteny was exploited to develop and saturate the susceptibility region with markers, delimiting it to ∼0.24 cM and a partial physical map was constructed. This genetic and physical characterization further resolved the dominant susceptibility locus, designated *Spt1* (susceptibility to *P. teres* f. *teres*). The high-resolution mapping and cosegregation of the *Spt1.R* and *Spt1.K* gene/s indicates tightly linked genes in repulsion or alleles possibly targeted by different necrotrophic effectors. Newly developed barley genomic resources greatly enhance the efficiency of positional cloning efforts in barley, as demonstrated by the *Spt1* fine mapping and physical contig identification reported here.

The necrotrophic fungus *Pyrenophora teres* is the causal agent of net blotch of barley (*Hordeum vulgare*) and exists in two forms which, although morphologically indistinguishable under the microscope, can be differentiated by the symptoms observed on susceptible barley genotypes (McLean *et al.* 2009; Smedegård-Peterson 1971). The disease net form net blotch (NFNB) caused by *P. teres* f. *teres* is manifested by necrotic lesions with transverse and longitudinal striations, forming a net-like pattern of necrosis often accompanied by chlorosis (Mathre 1997). The disease spot form net blotch (SFNB) caused by *P. teres* f. *maculata* produces elliptical necrotic lesions surrounded by chlorosis (McLean *et al.* 2009; Smedegård-Peterson 1971). Both NFNB and SFNB are destructive foliar diseases, which can cause yield losses of 10–40% (Mathre 1997; Murray and Brennan 2010) and reduction in barley end use quality. Although similar fungicide applications and cultural practices can be used to manage these diseases, the preferred method of management is through host resistance (Mathre 1997). However, even though these two pathogens are closely related and genetically very similar, their host–pathogen genetic interactions are distinct (reviewed in Liu *et al.* 2011), thus they must be treated as different diseases when deploying genetic resistances.

Using globally diverse barley cultivars and *P. teres* f. *teres* isolates, several genetic studies have positioned both dominant and recessive resistance to NFNB at the centromeric region of barley chromosome 6H, suggesting the presence of multiple diverse resistance or susceptibility genes. It is hypothesized that these host genes interact with different *P. teres* f. *teres* effectors/avirulence proteins from diverse isolates resulting in the differential compatible or incompatible reactions observed in this pathosystem (Ho *et al.* 1996; Steffenson *et al.* 1996; Raman *et al.* 2003; Cakir *et al.* 2003; Ma *et al.* 2004; Manninen *et al.* 2006; Friesen *et al.* 2006a; Abu Qamar *et al.* 2008; St. Pierre *et al.* 2010). Abu Qamar *et al.* (2008) mapped two recessive resistance genes, which exist in repulsion, in a double haploid population derived from a cross between the barley lines Rika and Kombar. Rika is resistant to *P. teres* f. *teres* isolate 15A and susceptible to *P. teres* f. *teres* isolate 6A, while Kombar is resistant to *P. teres* f. *teres* isolate 6A and susceptible to *P. teres* f. *teres* isolate 15A. The susceptibility loci were mapped to an ∼5.9 cM region near the centromere on barley chromosome 6H. The region was further delimited to an ∼3.3 cM interval through the use of EST-based markers (Liu *et al.* 2010).

A comprehensive genetic analysis utilizing a biparental population of *P. teres* f. *teres* isolates 6A and 15A, which exhibit reciprocal virulence on Rika and Kombar, respectively, identified four virulence QTL in the pathogen. Two of the QTL, designated *VR1* and *VR2* for virulence on Rika 1 and 2, were identified for *P. teres* f. *teres* isolate 6A and two QTL, *VK1* and *VK2*, for virulence on Kombar 1 and 2, were identified for *P. teres* f. *teres* isolate 15A (Shjerve *et al.* 2014). It was postulated that host selective toxins or necrotrophic effectors underlie these virulence QTL as it was recently shown in this pathosystem that a proteinaceous effector from *P. teres* f. *teres* isolate 0-1, designated PttNE1, interacts with a single dominant susceptibility gene on chromosome 6H in barley cv. Hector, inducing necrotrophic effector triggered susceptibility (NETS) (Liu *et al.* 2015). Also, other closely related necrotrophic ascomycete fungi, such as *Pyrenophora tritici-repentis* and *Parastagonospora nodorum*, produce necrotrophic effectors that alter the host’s innate immunity responses resulting in enhanced disease (Tomas *et al.* 1990; Strelkov *et al.* 1999; Liu *et al.* 2009, 2012; Gao *et al.* 2015). The proteinaceous necrotrophic effector ToxA produced by *P. tritici-repentis* (Ptr ToxA) and *P. nodorum* (SnToxA), interact with the dominant host sensitivity protein Tsn1 (Friesen *et al.* 2006b; Faris *et al.* 2010). Tsn1 has protein domains similar to typical resistance proteins that confer resistance to biotrophic pathogens through the induction of tightly regulated programmed cell death (PCD) pathways. These pathways are typically thought to have evolved to sequester biotrophic pathogens which require living host tissues to complete their lifecycle. The inappropriate elicitation of these innate resistance pathways through the use of necrotrophic effectors by the necrotrophic pathogens allow them to subvert and utilize the host immunity pathways to infect and complete their lifecycle in the host. Thus, they utilize necrotrophic effectors to target specific host susceptibility genes, that otherwise represent immune receptors, to elicit NETS. These interactions between the host and pathogen are similar to H. H. Flor’s classical gene-for-gene paradigm (Flor 1956), yet are considered to be an inverse gene-for-gene relationship in which the specific interactions leading to PCD result in a compatible interaction (susceptibility) in a quantitative manner due to the nature of the pathogen’s lifestyle (Friesen *et al.* 2007). However, it also appears that classical gene-for-gene or dominant resistances also exist in this pathosystem as Friesen *et al.* (2006a) identified dominant resistance to three different NFNB isolates, which interestingly also mapped to the centromeric region of barley chromosome 6H.

Grass species, such as the globally important crops barley, wheat (*Triticum aestivum*), maize (*Zea mays*), rice (*Oryza sativa*), and sorghum (*Sorghum bicolor*), diverged from a common ancestor ∼55–70 million years ago (Kellogg 2001). Throughout their evolutionary history, it has been shown that the relative gene content and order have been conserved between the grass species, with much of the change in genome size being due to an increase of repetitive elements (Bennetzen and Freeling 1997; Kellogg 2001). Although synteny breaks attributed to chromosome insertions and translocations are observed, the overall gene order is well conserved (IBI 2010). The sequencing and annotation of the *Brachypodium distachyon* genome provided a useful tool for gene discovery in other grass species, especially crop species with large genomes as the result of expansion of repetitive DNA elements. These large repetitive genomes complicate genome sequence assembly such that there is adequate assembly of low copy gene spaces but many gaps at repeat regions, thus robust genome assemblies have not been achieved (Mur *et al.* 2011; IBGSC 2012). Syntenic conservation between the model grass genome sequence and barley and wheat expedites the development of markers in the large grass genomes allowing for the rapid saturation of regions for positional cloning (IBI 2010; Mur *et al.* 2011)

The draft assembly of the barley gene space was recently completed, however, a nearly complete genome assembly of the ∼5.1 Gb genome, which consists of approximately 84% repetitive elements, is a challenging task with the current bioinformatic software, but a draft physical map of the barley genome has been assembled via high-information content fingerprinting of bacterial artificial chromosome (BAC) clones and incorporation of whole-genome shotgun (WGS) sequence, BAC end sequence (BES), sequence from selected BAC clones, and integration of genetic mapping data (IBGSC 2012). Furthermore, additional anchoring of WGS contigs was accomplished through population sequencing (POPSEQ) and BAC clones have been assembled into minimum tiling paths across assembled contigs (Mascher *et al.* 2013; Ariyadasa *et al.* 2014) Additionally, 15,622 cv. Morex BACs were sequenced and subsequently integrated with consensus map and synteny analyses into the easily accessible software HarvEST (Muñoz-Amatriaín *et al.* 2015). The use of this resource allows for the physical mapping of regions identified by genetic map-based cloning strategies, which facilitates the identification of candidate genes in a more efficient manner.

The dominant susceptibility genes *Spt1.R/Spt1.K* (formerly *rpt.k/rpt.r*), which are hypothesized to confer dominant susceptibility to *P. teres* f. *teres* isolates 6A and 15A, appear to operate in a complex fashion. We hypothesize that alleles of *Spt1*, *Spt1.R*, and *Spt1.K*, or several tightly linked genes, confer major effect dominant susceptibility to *P. teres* f. *teres* isolates 6A and 15A, respectively. Here, we describe the high-resolution mapping of a dominant susceptibility locus located in the centromeric region of barley chromosome 6H. Mapping was done utilizing markers developed via the exploitation of the syntenic relationship with *B. distachyon* chromosome 3. Additionally, the identification of barley physical contigs, enabled by the newly developed barley genomic resources, were used to construct a partial physical map of the region. The results of this research will soon facilitate the identification of the dominant susceptibility gene *Spt1* and allow for the subsequent functional analysis of its alleles that are hypothesized to function by NETS. This research will fill gaps in knowledge concerning these complex host–parasite genetic interactions and the underlying molecular mechanisms determining compatible interactions in this complex barley-NFNB pathosystem.

## Materials and Methods

### Biological materials

The two *P. teres* f. *teres* isolates 6A and 15A, and progeny identified as containing single virulence QTL for VK1, VK2, and VR2 (Shjerve *et al.* 2014) from the cross between isolates 6A and 15A, were utilized for phenotyping. The *P. teres* f. *teres* isolate 6A was collected from Fresno County, California (Wu *et al.* 2003) and isolate 15A was collected from Solano County, California (Steffenson and Webster 1992).

The Scandinavian two-rowed barley variety Rika and US six-rowed barley variety Kombar were previously identified as having reciprocal reactions to *P. teres* f. *teres* isolates 6A and 15A. Rika is susceptible to *P. teres* f. *teres* isolate 6A and resistant to *P. teres* f. *teres* isolate 15A. Kombar exhibits an opposite reaction, being susceptible to *P. teres* f. *teres* isolate 15A and resistant to *P. teres* f. *teres* isolate 6A (Abu Qamar *et al.* 2008). Rika and Kombar were crossed and advanced to generate a population of 1488 F_2_ individuals, representing 2976 gametes, that were subsequently genotyped to identify recombinant gametes within the *Spt1* region. Previously identified critical recombinant double haploid (DH) progeny identified from a population consisting of 118 individuals derived from a Rika and Kombar cross (Abu Qamar *et al.* 2008) were also genotyped to validate the DH recombinants within the NFNB *Spt1* region, which was previously referred to as the *rpt.k* and *rpt.r* recessive resistance genes.

### Genotyping and recombinant gamete identification

Two previously identified flanking simple sequence repeat (SSR) markers, Bmag0173 on the distal side and Rbah21g15 on the proximal side (Liu *et al.* 2010), were used to genotype the 1488 Rika × Kombar F_2_ individuals. The DNA isolation for the initial F_2_ genotyping was performed at the US Department of Agriculture-Agricultural Research Service (USDA-ARS) cereal genotyping center, Fargo, ND, using the high-throughput procedure described by Tsilo *et al.* (2009).

After the F_2_ individuals with recombination within the *Spt1* region were identified from the initial round of genotyping with the SSR markers on the ABI sequencer, the recombinant seedlings were transplanted from conetainers into 6 inch pots, grown in the greenhouse, and allowed to self to produce F_2:3_ seed. Fifteen F_2:3_ plants from each family were grown in the greenhouse and genomic DNA isolated from approximately 2 cm long leaf tissue samples collected in 1.5 ml Eppendorf tubes. The leaf tissue was homogenized in 400 µl of extraction buffer (200 mM Tris-HCl pH 7.5, 250 mM NaCl, 25 mM EDTA, 0.5% SDS) with a disposable tissue grinder followed by the addition of 200 µl of chloroform. Samples were vortexed for 10 sec and centrifuged at 15,600 × *g* for 10 min. The supernatant (∼300 µl) was transferred to a new 1.5 ml tube and 300 µl of isopropanol was added. The samples were mixed by inversion and incubated at room temperature for 5 min followed by centrifugation at 15,600 × *g* for 10 min. The pellets were rinsed with 70% ethanol, dried, and resuspended in 100 µl of ddH_2_O. The 15 F_2:3_ individuals from each F_2_ family identified with a critical recombination in the *Spt1* region were genotyped utilizing the codominant marker rpt-M4 to identify homozygous recombinant individuals, which represent immortal critical recombinants (ICR). The ICR individuals were given a progeny designation and were utilized for all further phenotyping and genotyping.

### STS/SNP marker development

Utilizing the syntenic relationship between barley chromosome 6H and *B. distachyon* chromosome 3, genetic markers were developed to saturate the *Spt1* region through the use of the annotated *B. distachyon* genome sequence (IBI 2010). Orthologous loci in *B. distachyon* corresponding to the distal flanking EST marker BE636841 and the proximal SSR marker Rbah21g15 were used to identify the ∼1.02 Mb of syntenic *B. distachyon* genomic DNA sequence. Orthologous barley expressed sequence tags (ESTs) were identified, utilizing Blastn searches limiting to ESTs other_*Hordeum vulgare* that correspond to *B. distachyon* genes within the region. A set of these ESTs were selected at ∼60 kb intervals using predicted function as the criteria for selection, as certain genes have a higher probability of containing polymorphisms compared to others. For example, NBS-LRR genes typically have more diversity than highly conserved house-keeping genes. Oligonucleotide primers were designed from the barley EST sequences and used to produce amplicons from Rika and Kombar genomic DNA by PCR (Table 1). Genomic DNA was extracted as previously described. The parameters for the PCR reactions were as follows: 94° for 5 min, followed by 35 cycles of 94° for 30 sec, 62° for 30 sec, and 72° for 60 sec, followed by a final extension at 72° for 7 min. The PCR reactions consisted of 1.25 units NEB Standard Taq polymerase, forward and reverse primers (1.2 µM), NEB Standard Taq buffer (1 ×), and dNTPs (200 µM), in 25 µl reactions. PCR products were visualized on a 1% agarose gel containing GelRed (Biotium, CA) and subsequently purified using E.Z.N.A. Cycle Pure Kit (Omega Bio-Tek, Norcross, GA) following the manufacturer’s standard protocol. The amplicons were sequenced (McLab and Genscript) and single nucleotide polymorphisms (SNPs) were identified by alignment using Vector NTI software (Invitrogen) and NCBI nucleotide sequence alignment tools. Sequence tagged site (STS) markers were developed from the SNPs by designing genotype specific primers with SNP specific nucleotides corresponding to the 3′ terminal nucleotide of the primers (Table 1). If a robust STS marker was unable to be developed, the critical recombinants were amplified using the original primer pairs and the amplicons were Sanger sequenced (Genscript).

**Table 1:** Primer sequences (5′–3′) for polymorphic markers developed from barley orthologs of *Brachypodium distachyon* genes within the *Spt1* region

Marker	Class	Forward Primer	Reverse Primer
rpt-M4-1*^a^*	STS	AGGAAATGGTCTCTCCAAAGTCTC	ACATCTCATCATCTGGCCACCATAC
rpt-M4-2*^a^*	STS	TGAAGAGGAAGCGTGAACAAGATAG	TGACTACGAATGAATACCTCTTCAG
rpt-M5*^b^*	STS	AAAGAAGATCAGGCTTACCAGCATC	ATGCGACAACCAGGTAAGTAGAGTG
rpt-M8	SNP	GCTGGCCTCCAGCTTCGACGTGATG	AACGTAAGCTCATTCTACATAAGAC
rpt-M12.r*^b^*	STS	ATGGCCAACCAGCTTAAATATCCCA	TCACTCATGCAGAGTGGCGTACACCA
rpt-M12.k*^c^*	STS	GTGCCTACTTCTCTGTATATTCACG	GTCTCATTGCATGCGCTGTCACCTC
rpt-M13	STS	GCAGAACTCTACCAGCACTTCAGAG	CTTCCAGATGATTCAGGTTCATTAC
rpt-M20	SNP	CCATCAATACAGTGTTTATCACCAA	GCTCAAAATGTCCACAGTATTATCC
rpt-M32	SNP	ATGTATGGTAAATGTGGGGGTATC	GAGTAAATCAACCATTAGGCCATAG
rpt-M61	SNP	ACCTTTCGCCACCAACAACACAGAC	GTATGATGTCAAGCTGAACAATGCC
rpt-M62	Indel	GAATTAGCAGGAGACCAATGTAAGA	TTTCTTATTAGGTGATGCGTTTGTT
rpt-M14	SNP	AAGCACCACCCTGGACAGATAGAAG	ATGAGATCAGACCAAGTGAGTTCAC

STS, sequence tagged site; SNP, single nucleotide polymorphism; indel, insertion or deletion of bases in the DNA of an organism.

a Codominant marker when multiplexed in a single PCR reaction.

b Rika specific.

c Kombar specific.

### PCR-GBS library preparation and ion torrent sequencing

A PCR genotyping-by-sequencing (PCR-GBS) panel was also constructed consisting of two previously designed SNP markers from the T3 database (https://triticeaetoolbox.org/barley/) and 11 primer pairs designed from the predicted coding, intron, or 3′ UTR sequence from barley orthologs of *B. distachyon* genes within the region with the aim of SNP discovery. Additionally, four previously designed STS markers were included in the panel for regenotyping and internal control. A 22 nucleotide CS1 adaptor (5′-ACACTGACGACATGGTTCTACA-3′) (Fluidigm) was added to the 5′ end of forward primers and a 22 nucleotide CS2 adaptor (5′-TACGGTAGCAGAGACTTGGTCT-3′) (Fluidigm) was added to the 5′ end of the reverse primers. Barcoded adaptor primers were designed containing the Ion Torrent A adaptor sequence, unique 12 nucleotide barcode, and the CS1 adaptor sequence (5′-CCATCTCATCCCTGCGTGTCTCCGACTCAG(NNNNNNNNCGAT)ACACTGACGACATGGTTCTACA-3′). A universal reverse primer was designed containing the Ion Torrent P1 adaptor sequence and the CS2 adaptor sequence (5′- CCACTACGCCTCCGCTTTCCTCTCTATGGGCAGTCGGTGATTACGGTAGCAGAGACTTGGTCT-3′). Primers were multiplexed by adding 5 µl of each forward and reverse primer (100 µM) into a sterile 1.5 ml Eppendorf tube and 820 µl of nuclease-free H_2_O for a final primer pool volume of 1 ml. PCR reactions consisted of 1.5 µl genomic DNA, 1 µl of primer pool (100 nM each primer), and 2.5 µl Platinum Multiplex PCR Master Mix (Life Technologies). DNA concentrations were normalized to ∼20 ng/µl and 1.5 µl of each sample was pipetted into individual wells on a 96-well plate. Replicates of Rika, Kombar, RK107, RK121, and RK122 were also included on the plate. The primary PCR reaction was conducted as follows: Initial denaturation at 94° for 10 min, 10 cycles of 94° for 20 sec and 64° decreasing each cycle by 0.8° for 60 sec, followed by 20 cycles of 94° for 20 sec, 57° for 60 sec, and 68° for 30 sec, ending with a final extension of 72° for 3 min. The PCR plate was then briefly centrifuged and 15 µl of nuclease-free H_2_O was added to each sample. The plate was then sealed, vortexed, briefly centrifuged, and 2 µl of each sample was aliquoted into a new 96-well plate. Additionally, 2 µL of each reaction was separated on a 1% agarose gel containing GelRed to ensure successful reactions. The barcoding PCR reaction consisted of 0.235 µl of H_2_O, 0.625 µl NEB Standard Taq Buffer (1.25 ×), 0.1 µl dNTPs (500 µM), 1 µl universal reverse primer (1 µM), 1 µl unique barcode adaptor primer (0.4 µM), 0.04 µl of NEB Taq polymerase (0.2 units), and 2 µl template (diluted primary PCR reaction). PCR parameters are the same as the primary reaction. Following PCR, the plate was briefly centrifuged and each sample was diluted by adding 15 µl of H_2_O. Samples were pooled by aliquoting 5 µl of each sample into a 1.5 ml Eppendorf tube and purified using the E.Z.N.A. Cycle Pure Kit. The pooled library was then amplified to prepare for sequencing. PCR reactions consisted of a 2 µl sequencing library, 13 µl H_2_O, 2.5 µl NEB Standard Taq Buffer (1 ×), 3 µl ABC1 primer (0.6 µM), 3 µl P1 primer (0.6 µM), 0.5 µl dNTPs (200 µM), and 1 µl NEB Taq polymerase (5 units). PCR parameters were as follows: initial denaturation at 95° for 5 min, followed by 8 cycles of 94° for 30 sec, 62° for 30 sec, and 72° for 30 sec, ending with a final extension at 72° for 7 min. The amplified library was quantified using the Qubit dsDNA High Sensitivity Kit and the Qubit Fluorometer (Life Technologies) and diluted to ∼3 pg/µl. Sequencing was conducted on the Ion Torrent PGM utilizing the Ion PGM Template OT2 200 Kit, Ion PGM Sequencing 200 Kit v2, and an Ion 314 Chip (Life Technologies).

### SNP calling

Sequencing reads were trimmed by 22 nucleotides on the 5′and 3′ ends to remove the PCR adaptors. Trimmed reads were aligned to a reference file consisting of previously identified amplicon sequences from the T3 database, orthologous barley EST sequence corresponding to select *B. distachyon* genes within the region, and sequence from previously designed STS markers using the BWA-MEM algorithm with default settings (Li and Durbin 2009), and the alignments were converted to BAM files using SAMtools (Li *et al.* 2009). SNPs were identified using the Genome Analysis Toolkit’s Unified Genotyper tool (DePristo *et al.* 2011). Called SNPs were then filtered based on the parameters of a minimum genotype quality of 10 and a minimum read depth per site per individual of 3.

### Phenotyping

The ICRs were phenotyped with *P. teres* f. *teres* isolates 6A, 15A, and the progeny isolates 15 × 6A #20, 15A × 6A #63, and 15A × 6A #72, with the Mendelized virulence QTL *VK1*, *VK2*, and *VR2*, respectively. Phenotyping was conducted as described by Friesen *et al.* (2006a). Briefly, inoculum was prepared by plating agarose plugs of the *P. teres* f. *teres* isolates on V8 PDA media (150 ml V8 Juice, 10 g Potato Dextrose Agar, 10 g agarose, 3 g CaCO_3_, and 850 ml H_2_O). Fungal cultures were incubated at room temperature in the dark for 5 d, placed under light at room temperature for 24 hr, and then returned to the dark at 15° for 24 hr. Conidia were harvested by flooding the plates with H_2_O and scraping the culture with a sterile inoculation loop. Inoculum was adjusted to a concentration of ∼2000 spores/ml and one drop of Tween 20 was added per 50 ml of inoculum. Three seeds of each ICR line were planted in a single conetainer (Stuewe and Sons, Inc., Corvallis, OR.) and three conetainers were planted for each ICR, totaling nine seedlings per line. Seedlings were grown under greenhouse conditions until the second leaf was fully expanded, approximately 14 d. Inoculum was applied as described by Friesen *et al.* (2006a) and incubated at 21° and 100% relative humidity for 22–24 hr. Plants were then moved to a growth chamber at 21° with a 12 hr photoperiod and were scored 7 d post inoculation using a 1–10 scale (Tekauz 1985). Nine plants per line were scored collectively as one experimental unit and inoculations were replicated three times.

### High-resolution map construction

A total of 14 ICRs harboring a recombination between flanking markers Bmag0173 and Rbah21g15 were identified and utilized in the construction of a high-resolution map. Genetic distances were calculated based on recombination frequency between markers and denoted as map units (M.U.).

### Barley iSELECT 9K chip

The barley *9k* Illumina *Infinium* iSELECT assay was utilized to genotype the parents Rika and Kombar and eight of the ICR lines (RK122, 125, 130, 139, 151, 173, 183, and 188) with recombination in the *Spt1* region. The genotyping was performed as described by Muñoz-Amatriaín *et al.* (2014).

### BAC library screening and physical map construction

Utilizing the current barley physical map (IBGSC 2012) containing anchored WGS and BAC sequences, as well as the Morex 6H minimum tiling path (MTP) BAC library (Mascher *et al.* 2013; Ariyadasa *et al.* 2014), genetic markers from the high-resolution map were anchored to physical contigs. Sequences of the markers within the region were used in BLAST searches of the Morex WGS database to identify positive WGS contigs (http://webblast.ipk-gatersleben.de/barley/viroblast.php). When possible, positions of the WGS contigs on larger fingerprinted contigs (FPCs) were identified. Those markers that did not yield anchored WGS contigs were used to screen the Morex 6H MTP BAC library via PCR (INRA-CNRGV) to identify its position on a FPC. Additionally, a Morex BAC library (Yu *et al.* 2000) was screened with markers rpt-M8, rpt-M12, and rpt-M13, as described by Brueggeman *et al.* (2002). Positively identified BAC clones were plated on LB plates containing chloramphenicol (50 µg/ml) and incubated overnight at 37°. Colonies were then inoculated into liquid LB containing chloramphenicol (50 µg/ml) and incubated for 16 hr at 37° with shaking at 230 rpm. BAC DNA was isolated as described in Brueggeman *et al.* (2002). PCR was conducted on BAC DNA with the markers rpt-M8, rpt-M12, and rpt-M13 to identify corresponding BAC clones using the aforementioned PCR protocol. Additionally, the HarvEST database, which houses BAC sequences from the Yu *et al.* (2000) library, was BLAST searched with marker sequences to identify and confirm additional BAC clones (Muñoz-Amatriaín *et al.* 2015).

### BAC library sequencing and assembly

DNA from the selected BACs 783N21 and 650G13 (Yu *et al.* 2000) was isolated as previously described. BAC DNA was pooled and sequenced at the Washington State University Molecular Biology and Genomics Core using Ion Torrent PGM technology. Sequencing reads were *de novo* assembled using a CLC Bio Genomics Workbench (Qiagen) requiring 95% sequence homology and minimum contig size of 1 kb for assembly.

### Data availability

Data and reagents are available upon request. Supplemental Material, File S1 contains genotypes of the ICRs from the newly developed markers. File S2 contains the raw phenotypic data for Rika, Kombar, and the ICRs from inoculations with the aforementioned *P. teres* f. *teres* isolates.

## Results

### Initial genotyping and phenotyping of critical recombinants

A total of 1488 F_2_ individuals and 118 DH lines derived from a cross of Rika × Kombar were genotyped with the flanking SSR markers Bmag0173 and Rbah21g15. Thirteen F_2_ individuals and one DH line were identified that contained recombination within the previously delimited *rpt.r/rpt.k* region, now referred to as the *Spt1* region. Subsequently, the F_2:3_ families of these 13 critical recombinants were genotyped with the codominant marker rpt-M4 and an immortal critical recombinant (ICR) individual was identified for each F_2:3_ family.

### Phenotyping of the ICR lines

Individuals from each ICR family and the lone DH individual were then phenotyped with *Pyrenophora teres* f. *teres* isolates 15A, 6A, 15 × 6A #20, 15A × 6A #63, and 15A × 6A #72. Lines that exhibited an average disease reaction of five or less were classified as resistant and lines that displayed average reactions greater than five were considered susceptible. Following inoculations with isolate 15A, Rika and Kombar exhibited average reactions of 1.67 and 7.5, respectively. A total of five ICRs were classified as resistant with individual averages ranging from 1–3.33 and an overall average of 1.77. The remaining nine ICRs exhibited individual average disease reactions ranging from 6.83–7.83 with an overall average of 7.59 and were classified as susceptible (Table 2). When inoculated with progeny isolate 15A × 6A #20 (Shjerve *et al.* 2014) containing only the *VK1* virulence locus, Rika and Kombar exhibited average disease reactions of 1.83 and 6.83, respectively. Five ICRs were considered resistant and nine classified as susceptible. The average disease reaction of the resistant lines ranged from 1.83–3.83 with an overall average of 2.73. For the susceptible group, individual averages ranged from 6.67–8.83 with an overall mean of 7.74 (Table 2). Phenotypic data from inoculations with a second progeny isolate, 15A × 6A #63, harboring only the *VK2* virulence locus (Shjerve *et al.* 2014) showed the same pattern. Disease reaction averages for Rika and Kombar were 2.17 and 7.17, respectively. The previously classified resistant and susceptible ICRs grouped together again. The disease score averages of resistant individuals ranged from 1.5–2 with an overall mean of 1.77. Susceptible individuals exhibited average disease scores ranging from 5.33–7.17 and an overall average of 6.35 (Table 2).

**Table 2:** Average disease scores for Rika, Kombar, and resistant/susceptible ICRs with *Ptt* isolates

	Average Reaction Type				
Genotype	15A	15A × 6A #20 (VK1)	15A × 6A #63 (VK2)	6A	15A × 6A #72 (VR2)
Rika	1.67 ± 0.58	1.83 ± 0.29	2.17 ± 0.29	9.5 ± 0.50	7.83 ± 1.04
Kombar	7.5 ± 0.50	6.83 ± 1.76	7.17 ± 1.44	4.17 ± 1.26	1 ± 0
Resistant ICRs	1.77 ± 0.92	2.73 ± 0.89	1.77 ± 0.25	4.06 ± 0.61	1.15 ± 0.18
Susceptible ICRs	7.59 ± 0.35	7.74 ± 0.83	6.35 ± 0.63	7.65 ± 1.62	7.97 ± 0.71

ICR, immortal critical recombinants.

Following inoculations of the ICRs with isolate 6A, Rika and Kombar displayed reaction types of 9.5 and 4.17, respectively. Average disease reaction types for six resistant lines ranged from 3.5–5 with an overall mean of 4.06. A total of eight individuals were considered susceptible and average reaction types ranged from 5.50 to 9.33 with an overall average of 7.65 (Table 2). After inoculation with progeny isolate 15A × 6A #72 harboring only the *VR2* virulence locus (Shjerve *et al.* 2014), Rika and Kombar showed average reaction types of 7.83 and 1, respectively. A total of nine ICRs were considered resistant with average disease scores ranging from 1.00–1.50 and a total mean of 1.15. The susceptible class of ICRs consisted of five individuals with average disease ratings ranging from 7.17–8.67 and an overall average of 7.97 (Table 2). Several individuals (RK121, RK122, and RK173) exhibited intermediate reactions ranging from 5.50–6.17 when inoculated with isolate 6A and subsequently were classified as susceptible. However, due to the averages of these three individuals being near the mean score of Kombar and the 6A resistant individuals, as well as these individuals being highly resistant to isolate 15A × 6A #72 (VR2), these apparent intermediate reactions may not accurately represent the classification of susceptible.

### Marker development and high-resolution map construction

**Figure 1:**
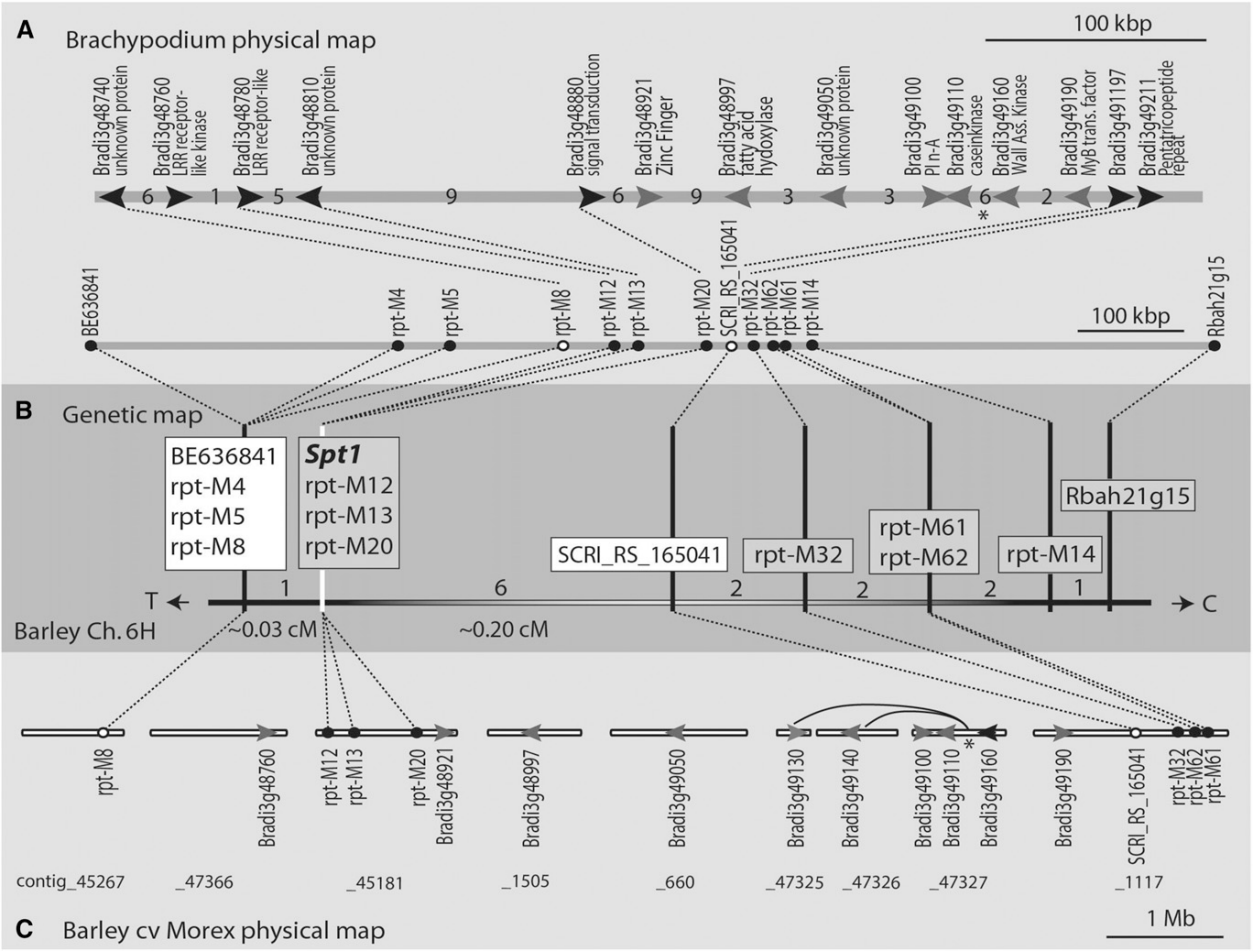
Genetic and partial physical map of the *Spt1* locus in synteny with *Brachypodium distachyon*. Syntenous *B. distachyon* sequence, Rika × Kombar barley high-resolution map and barley cv. Morex physical map at the *Spt1* locus. (A) The gray horizontal bars represent the syntenous *B. distachyon* chromosome 3 sequence delimiting the *Spt1* gene region from barley. The lower bar shows the relative position of barley orthologous sequences used to develop markers from the annotated *B. distachyon* genes. Black dots represent marker positions and white dots represent the *Spt1* flanking markers. The upper bar is an expanded and more detailed representation of the delimited *Spt1* region with annotated *B. distachyon* genes shown with arrows and labeled above with putative gene functions. The black arrows represent genes from which barley orthologs were used to generate SNP (single nucleotide polymorphism) markers and the gray arrows are genes that did not have polymorphisms in the Rika × Kombar population but were utilized to anchor physical contigs in panel C. The numbers between arrows are the number of additional *B. distachyon* genes present between each gene designated with an arrow. The 100 kb scale is shown above and below. (B) Rika × Kombar chromosome 6H (Ch.6H) *Spt1* high-resolution map generated by screening 2976 recombinant F_2_ gametes. The boxes contain cosegregating markers with white boxes containing markers that delimit the *Spt1* locus. Numbers between the vertical lines connected to the marker boxes indicate the number of recombinants between the markers. T and C at the end of the horizontal line indicate the direction of telomere and centromere, respectively. (C) Barley cv. Morex physical map showing the relative sizes of the supercontigs (white horizontal bars) with approximated positions of genetic markers generated from *B. distachyon* orthologous barley genes. The black circles indicate position of markers with the white circles indicating the *Spt1* region flanking markers. The position of the barley genes identified by *B. distachyon* orthologs that we were unable to develop markers from but could anchor to the barley physical map are indicated by gray arrows. The proximal flanking SNP marker SCRI_RS_165041, identified from the barley *9k* Illumina *Infinium* iSELECT assay position, is also designated with a white circle. The asterisk indicates two super contigs that were out of synteny between *B. distachyon* and barley. Barley contigs_47325 and _47326 were identified to contain barley orthologs of the annotated *B. distachyon* genes Bradi3g49130 and Bradi3g49140, which in the *B. distachyon* sequence are located between Bradi3g49110 and Bradi3g49160. The cv, Morex supercontig designations (IBGSC, 2012) are provided below with the 1 Mb scale.

Utilizing synteny between barley and *B. distachyon*, orthologous *B. distachyon* genes Bradi3g48220.1 and Bradi3g49360.1 corresponding to the *Spt1* flanking markers BE636841 and Rbah21g15, respectively, were identified via BLAST searches. This approximately 1.02 Mb *B. distachyon* genomic region between the two orthologs harbors 149 annotated genes. *B. distachyon* genes containing syntenic barley orthologs at approximately 60 kb intervals were selected and used in BLAST searches to identify high-confidence barley unigenes with EST support. Primers were designed from the 16 identified unigenes and used to produce gene specific amplicons from the barley cultivars Rika and Kombar. Sanger sequencing was performed on the amplicon and sequence alignments used to characterize allelic differences. Sequencing of the 16 selected unigenes yielded six robust amplicons containing at least one SNP between the parents. Allele specific primers with 3′ terminal nucleotides specific to each parental SNP were designed and used as STS markers to genotype the ICRs to saturate the high-resolution map with markers at the *Spt1* locus. STS markers were unable to be developed from SNPs within two barley orthologs, designated as rpt-M8 and rpt-M14. The original primer pairs were used to produce amplicons from each ICR and subsequently Sanger sequenced for genotyping. Markers rpt-M4, rpt-M5, and rpt-M8 cosegregated and mapped distal to the susceptibility locus, rpt-M12 and rpt-M13 cosegregated with *Spt1*, and rpt-M14 mapped proximal to the susceptibility locus. The newly developed markers delimited the *Spt1* locus to ∼0.43 cM between the distal flanking marker rpt-M8 and the proximal flanking marker rpt-M14. The delimited region in barley corresponded to ∼540 kb of *B. distachyon* sequence which contained 69 annotated genes. Using the aforementioned process of marker development via synteny, focus was then placed on the further saturation of the region. A total of 17 orthologous barley unigenes were identified from *B. distachyon* genes within this region and targeted for marker development, of which allele sequencing further identified two orthologs containing SNPs. The newly identified markers, rpt-M20 and rpt-M61, were subsequently genotyped via PCR directed genotype-by-sequencing of target amplicons due to the inability to develop efficient STS markers. Additionally, a robust indel marker, rpt-M62, was developed from the same ortholog as rpt-M61. Data procured from the barley iSelect 9K chip also revealed a SNP marker, SCRI_RS_165041, which further delimits the region. Currently, the *Spt1* gene is delimited by markers rpt-M8 and SCRI_RS_165041 to an ∼0.24 cM region. (Figure 1). The ∼0.24 cM region delimiting *Spt1* in barley corresponds to ∼466 kb of *B. distachyon* sequence harboring 62 annotated genes, and is predicted to contain 49 barley genes using anchoring data from the barley genome database and POPSEQ data, as well as BLAST searches of the barley genome using *B. distachyon* genes as the query. Attempts to utilize these genes to further saturate the genetic map identified 15 orthologous barley genes within the region. However, allele sequencing of introns, 3′ UTRs, and coding regions from Rika and Kombar of these select orthologs determined that these genes did not contain polymorphisms in our population (Figure 1)

### PCR-GBS sequencing and SNP calling

Sequencing on the Ion Torrent PGM platform resulted in a total of 569,040 reads averaging 82 bp in length. However, due the presence of primer dimers, only 184,174 reads were amplicon specific which averaged to ∼5581 reads per individual, which is sufficient to call SNPs for the amount of markers in this panel. Amplicons within the library were represented to a varying degree due to the differences in PCR efficiency. The two SNP markers from the T3 database (11_21216 and 11_20651) were not polymorphic between Rika and Kombar. However, we were able to identify polymorphism between Rika and Kombar within the amplicon of a single selected ortholog of a *B. distachyon* gene within the region, designated rpt-M32. The SNP marker rpt-M32 mapped proximal to SCRI_RS_165041 and distal to rpt-M14 (Figure 1). Additionally, two of the four previously identified STS markers (rpt-M4 and rpt-M8) had enough representative sequence to call SNPs and confirmed previous genotyping.

### BAC library screening and physical map construction

Screening of a barley cultivar Morex BAC library with markers rpt-M8, rpt-M13, and rpt-M12 identified four BAC clones and were confirmed via PCR (Table 3). Marker rpt-M8 did not amplify from any of the identified BAC DNA templates. The markers rpt-M12 and rpt-M13 were both amplified from the 783N21, 302J12, and 082C13 BAC DNA templates, indicating that these BAC clones comprise a MTP in which both markers reside. Additionally, via BLAST searches on the HarvEST database (Muñoz-Amatriaín *et al.* 2015), the location of markers rpt-M13 and rpt-M12 on BAC clone 783N21 was confirmed, as well as identified an additional BAC, 650G13, corresponding to marker rpt-M12. All the markers used in genotyping the high-resolution mapping population were also BLAST searched utilizing the HarvEST server to identify the BACs from the Yu *et al.* (2000) library on which they reside, resulting in the identification of corresponding BAC clones for each marker, except rpt-M8 and rpt-M14 (Table 3). Also, the marker sequences were BLAST searched using the IPK Barley BLAST server to identify corresponding sequenced BAC clones, cv. Morex WGS contigs, and, if possible, position on larger FPCs. Markers rpt-M8, rpt-M20, rpt-M32, and rpt-M14 were also used to screen the cv. Morex MTP BAC library (INRA-CNRGV) to successfully identify corresponding BAC clones and a position on a FPC due to the inability to anchor them to an FPC via BLAST search methods (Table 3).

**Table 3:** Bacterial artificial chromosome (BAC) clones identified from all markers on high-resolution map with corresponding sequence contigs and fingerprinted contigs

Marker*^a^*	Morex WGS Contig*^b^*	FPC Contig*^c^*	BAC Clone*^d^*	BAC Clone*^e^*
rpt-M4	morex_contig_38836	N/A	387C23, 442K9	N/A
rpt-M5	morex_contig_1564520	contig_4406	227HO5, 534F01, 729E01	N/A
rpt-M8	morex_contig_1573477	contig_45267	N/A	eA0312J23
rpt-M12	morex_contig_43862	contig_45181	783N21, 650G13, 82C13, 302J12	N/A
rpt-M13	morex_contig_64570	contig_45181	783N21, 82C13, 302J12	N/A
rpt-M20	morex_contig_37494	contig_45181	105J02, 606N13	hA0105J02, eA0192O20, mA0024H19
rpt-M32	morex_contig_102499	contig_1117	72B22, 154J12	mA0346B09, mA0406I23, hC0118K01
SCRI_RS_165041	morex_contig_1559439	contig_1117	575NO5	N/A
rpt-M61	morex_contig_45053	contig_1117	762H13, 344C02	mA0406I23
rpt-M62	morex_contig_45053	contig_1117	762H13, 344C02	mA0406I23
rpt-M14	morex_contig_52512	contig_6770	N/A	eA0049J16

WGS, whole-genome sequence; FPC, fingerprint contig; BAC, bacterial artificial chromosome; N/A, not applicable.

a The rpt designation refers to the recessive resistance nomenclature previously used in the literature.

b WGS contig from barley cultivar Morex identified via BLAST search (IBGSC 2012).

c FPC contig identified via barley physical map browser or BAC MTP (minimum tiling path) (IBGSC 2012; Ariyadasa *et al.* 2014).

d BAC clones identified from Morex BAC library (Yu *et al.* 2000) by hybridization and BLAST (Muñoz-Amatriaín *et al.* 2015).

e BAC clones identified from Morex 6H MTP BAC library, screened by Institut National de la Recherche Agronomique (INRA) or via BLAST search (BAC prefix HVVMRXALL) (IBGSC 2012).

## Discussion

Previous studies on host resistance to NFNB in barley have been conducted utilizing diverse arrays of barley lines, as well as fungal isolates from geographically distinct regions of the world. The results of these investigations were the identification of resistance/susceptibility loci distributed throughout the barley genome (Ho *et al.* 1996; Steffenson *et al.* 1996; Raman *et al.* 2003; Cakir *et al.* 2003; Ma *et al.* 2004; Manninen *et al.* 2006; Friesen *et al.* 2006a; Abu Qamar *et al.* 2008; St. Pierre *et al.* 2010). Although dominant susceptibility and/or resistance loci have been mapped to all seven barley chromosomes (reviewed by Liu *et al.* 2010), a common significant QTL near the centromere of chromosome 6H has repeatedly been associated with NFNB resistance and/or susceptibility. We have begun to characterize this important locus, and it is likely that the QTL region contains several closely linked genes that interact with the putative necrotrophic effectors underlying the four major virulence QTL identified in a *P. teres* f. *teres* 6A × 15A pathogen population (Shjerve *et al.* 2014). However, allele analysis of the candidate genes identified in this research suggests that underlying this major susceptibility locus is a single gene, with divergent alleles that confer dominant susceptibility to the California *P. teres* f. *teres* isolates 15A and 6A (J. Richards and R. Brueggeman, unpublished data).

The presence of multiple dominant susceptibility specificities (a.k.a. recessive resistance genes) at this locus (Abu Qamar *et al.* 2008; Liu *et al.* 2015) indicate that a repertoire of effector/virulence genes evolved in the pathogen to target a complex region of host biotrophic immunity receptors to elicit NETS. As seen in other fungal necrotrophic specialist pathosystems, the recessive resistance designation previously given to these host–pathogen genetic interactions are probably mediated by a functional dominant susceptibility gene product instead of a nonfunctional allele and should be more appropriately described as dominant susceptibility within an inverse gene-for-gene model (Friesen *et al.* 2007). Similar to the classic gene-for-gene paradigm (Flor 1956), a dominant gene product produced by the host interacts with a dominant gene product from the pathogen. However, due to the necrotrophic lifestyle of this specialist pathogen, recognition by the host results in a PCD immunity response that is subverted by the necrotroph, resulting in a compatible reaction and disease. It has been observed that several necrotrophic plant pathogenic fungi, such as members of the genera *Pyrenophora* and *Parastagonospora*, produce effectors, which are recognized by a single host gene (Friesen *et al.* 2006b; Faris *et al.* 2010). Recognition of necrotrophic effectors and subsequent cellular signaling by the host may occur via “resistance” genes encoding proteins with nucleotide binding site (NBS), leucine rich repeats (LRR), and/or serine-threonine protein kinase (STPK) domains (Lorang *et al.* 2007; Faris *et al.* 2010). Typical host resistance response mechanisms, such as the induction of programmed cell death, production of reactive oxygen species (ROS), and the up-regulation of PR genes, occur following host recognition of a necrotrophic effector (Lorang *et al.* 2007; Stergiopoulos *et al.* 2013; Liu *et al.* 2015). Although these responses provide effective defense against biotrophic pathogens, the necrotrophs that survive the hostile host induced environment produced as a barrier to colonization, also intentionally induce PCD immunity responses to obtain nutrients from the resulting dead host tissue. However, in contrast to biotrophic host resistance by these pathways that typically confer qualitative dominant resistances, these inverse gene-for-gene interactions appear to be quantitative in nature (Friesen *et al.* 2008).

Through high-resolution mapping of a Rika × Kombar F_2_ population utilizing markers derived from orthologous *B. distachyon* genes within the previously delimited *rpt.k/rpt.r* region, we have delimited a putative dominant susceptibility locus, designated *Spt1*, to an ∼0.24 cM region near the centromere of barley chromosome 6H. This high-resolution mapping greatly refined the previously delimited ∼3.3 cM region and facilitated the construction of a physical map using the new barley genome sequence. The use of the various newly available barley genomic resources, which are quickly being refined and updated, expedited the genetic to physical map construction and candidate *Spt1* gene identification. Although the physical map of the *Spt1* region is not completely contiguous, these tools have allowed for the positioning of physical sequences in correlation with our high-resolution map, as well as identification of flanking BAC clones to be used for further marker development and map saturation. Due to the identification of barley contigs containing the syntenic *B. distachyon* genes, we expect that we have captured the majority of the *Spt1* physical region and show that it has been delimited to ∼9.5 Mb of the barley genome which is predicted to contain 49 genes.

We hypothesize that the gene/s underlying the *Spt1* locus within the characterized region confers dominant susceptibility to NFNB isolates 6A and 15A through the recognition of necrotrophic effectors produced by the pathogen and the subsequent induction of PCD pathways in the host. It has recently been shown that *P. teres* f. *teres* produces necrotrophic effectors that induce the aforementioned host responses (Liu *et al.* 2015). Using intercellular wash fluids from barley leaf tissue inoculated with *P. teres* f. *teres* isolate 0-1, necrosis was induced on the susceptible cultivar Hector and sensitivity was subsequently mapped to the centromeric region of chromosome 6H. The segregation analysis indicated that a single locus at 6H likely contains a gene conferring sensitivity to an effector produced by *P. teres* f. *teres* isolate 0-1 (Liu *et al.* 2015). Additionally, four virulence loci, (*VR1*, *VR2*, *VK1*, and *VK2*), have been mapped in a *P. teres* f. *teres* mapping population derived from a cross of isolates 6A and 15A, identifying two major virulence QTL per isolate in different regions of the *P. teres* f. *teres* genome. Progeny isolates containing single virulence loci were identified for *VK1*, *VK2* and *VR2*, essentially Mendelizing the major virulence QTL from 15A and one from 6A by isolating the specific necrotrophic effector–host interactions. These single QTL progeny isolates were inoculated onto the 6H critical recombinants to map the sensitivity genes, which we expect will encode immunity receptor-like proteins that govern susceptibility mediated by these unique effectors. Susceptibility to the *VR2*, *VK1*, and *VK2* single major virulence progeny isolates mapped to the same region on 6H and, interestingly, all mapped to the *Spt1* locus. This indicates that virulence conferred by isolate 15A may be governed by the recognition of two unique necrotrophic effectors by two tightly linked genes. Alternatively, a single gene at the *Spt1* locus may perceive both necrotrophic effectors produced by isolate 15A. The putative necrotrophic effector produced by a progeny isolate harboring *VR2*, conferring virulence to isolate 6A, may also interact directly or indirectly with a dominant susceptibility factor at the *Spt1* locus, which may or may not be alleles of a gene conferring susceptibility to isolate 15A. Alternatively, several effector targets within the ∼0.24 cM *Spt1* genetic interval may exist in a “susceptibility island,” each recognizing a unique necrotrophic effector and eliciting PCD. We have delimited the genetic region to ∼9.5 Mb of barley physical sequence which, based on POPSEQ anchoring of barley unigenes and BLAST searches of orthologous *B. distachyon* genes within the region against the barley genome sequence, contains a predicted 39 high-confidence genes. Based on annotation of the functional domains, six of the genes encode predicted immunity receptor-like proteins. Thus, these genes are candidate *Spt1* genes and are being further analyzed via allele analysis and high-resolution association mapping.

The barley-NFNB pathosystem proves to be genetically complex, consisting of dominant, recessive, and incomplete resistances, which are clustered at the 6H locus. This 6H complexity complicates efforts to incorporate effective resistance into elite barley lines. The cloning of *Spt1*, as well as the effectors that interact directly or indirectly with them, will allow for greater knowledge and understanding of the molecular mechanisms underscoring this pathosystem, as well as how necrotrophic specialist fungal pathogens have evolved to exploit the intricate immunity system of the host to induce beneficial PCD responses. The isolation of individual resistance/susceptibility genes implicated in this pathosystem will fill gaps in the understanding of the molecular basis of necrotrophic specialist–host interactions and allow researchers to effectively utilize identified sources of resistance. This work greatly advances the effort toward the positional cloning and functional analysis of the host gene/s involved in this complex pathosystem.

## Supplementary Material

Supplemental Material
