# Supplementary material for: Fine Mapping of the Barley Chromosome 6H Net Form Net Blotch Susceptibility Locus
Source: G3 (Bethesda). 2016 Apr 19;6(7):1809–18. doi: 10.1534/g3.116.028902 (PMC4938636; doi:10.1534/g3.116.028902)
Supplement: Supplemental Material [file supp_6_7_1809__index.html]

Fine Mapping of the Barley Chromosome 6H Net Form Net Blotch Susceptibility Locus — Supplemental Material 

# Fine Mapping of the Barley Chromosome 6H Net Form Net Blotch Susceptibility Locus

## Supplemental Material for Richards *et al.*, 2016

**Files in this Data Supplement:**

- File S1 - Genotypic data of the immortal critical recombinants (ICRs). (.xlsx, 11 KB)
- File S2 - Phenotypic data. (.xlsx, 10 KB)
